# High rates of parkinsonism in adults with autism

**DOI:** 10.1186/s11689-015-9125-6

**Published:** 2015-08-30

**Authors:** Sergio Starkstein, Scott Gellar, Morgan Parlier, Leslie Payne, Joseph Piven

**Affiliations:** Fremantle Hospital, University of Western Australia, 35 Stirling Highway, Crawley, Western Australia Australia; Autism Association of Western Australia, Western Australia, WA 6008 Australia; School of Psychiatry, University of Western Australia, 35 Stirling Highway, Crawley, Western Australia Australia; Carolina Institute for Developmental Disabilities, School of Medicine, University of North Carolina at Chapel Hill, CB# 3367, Chapel Hill, NC 27599 USA

**Keywords:** parkinsonism, autism, movement disorders, parkinson’s disease, adults

## Abstract

**Background:**

While it is now recognized that autism spectrum disorder (ASD) is typically a life-long condition, there exist only a handful of systematic studies on middle-aged and older adults with this condition.

**Methods:**

We first performed a structured examination of parkinsonian motor signs in a hypothesis-generating, pilot study (study I) of 19 adults with ASD over 49 years of age. Observing high rates of parkinsonism in those off atypical neuroleptics (2/12, 17 %) in comparison to published population rates for Parkinson’s disease and parkinsonism, we examined a second sample of 37 adults with ASD, over 39 years of age, using a structured neurological assessment for parkinsonism.

**Results:**

Twelve of the 37 subjects (32 %) met the diagnostic criteria for parkinsonism; however, of these, 29 subjects were on atypical neuroleptics, complicating interpretation of the findings. Two of eight (25 %) subjects not taking atypical neuroleptic medications met the criteria for parkinsonism. Combining subjects who were not currently taking atypical neuroleptic medications, across both studies, we conservatively classified 4/20 (20 %) with parkinsonism.

**Conclusions:**

We find a high frequency of parkinsonism among ASD individuals older than 39 years. If high rates of parkinsonism and potentially Parkinson’s disease are confirmed in subsequent studies of ASD, this observation has important implications for understanding the neurobiology of autism and treatment of manifestations in older adults. Given the prevalence of autism in school-age children, the recognition of its life-long natural history, and the recognition of the aging of western societies, these findings also support the importance of further systematic study of other aspects of older adults with autism.

## Background

Autism spectrum disorder (ASD) is defined by the presence of characteristic behaviors present in early childhood [1]. While it is now recognized that ASD is typically a life-long condition, there exist only a handful of systematic studies of middle-aged and older adults [2–4]. According to the most recent estimate from the Centers for Disease Control (CDC), the prevalence of ASDs in US children is approximately 1.5 % [5]. Given the well-documented aging of the population in western societies [6], we and others have highlighted the need for increasing research efforts into all aspects of ASD in the aging population, to address what undoubtedly will be an important and increasing public health issue [7].

To begin to examine ASD in older (i.e., relative to previous studies of ASD) individuals with ASD, several years ago we began a pilot, descriptive study in North Carolina (NC) of adults with ASD 50 years and older. This study focused broadly across a range of domains—including current autistic behaviors, medical/psychiatric/neurological problems, cognitive level, adaptive behavior, quality of life, family/community support, and available services, to inform ourselves about specific areas for more concentrated focus. As the project progressed, we observed what appeared to be high rates of parkinsonian signs in our ASD subjects (study I). We, therefore, sought to ascertain a second sample of adults with ASD in which to conduct a systematic assessment of parkinsonian signs (study II) and examine the hypothesis generated from study I that “older” individuals with ASD had elevated rates of parkinsonian signs. We herein report the results from the examination of samples in studies I and II.

While systematic studies of Parkinson’s disease and parkinsonism have not been conducted in adults with ASD, a number of indirect and direct links between autism and Parkinson’s disease have been suggested in the research literature. Hollander et al. [8] suggested links between the two conditions based on overlapping phenomenology in the area of repetitive behaviors, with a common underlying involvement of the basal ganglia. Links between autism and the basal ganglia have been reported by a number of studies [9–11]. Recently, motor system deficits have been suggested as having a core role in ASD, based on the observation of motor dysfunction in children, adolescents, and young adults [12–17]. Motor deficits have been reported in infants prior to the typical time for diagnosis of ASD [18–21], suggesting the possible primacy of the motor system in autism. Overlap has also been noted in genetics, where the Park2 gene, implicated in Parkinson’s disease, has also been found in association with ASD [22]; Park2 copy number variant mutations have been identified in case reports of children with Asperger syndrome [23]; and mutations have been identified in a gene-regulating dopamine metabolism and behavioral responses to dopaminergic drugs, also implicated in Parkinson’s disease [24]. Establishing a precedent that genes linked to neurodevelopmental disorders also play a role in late-life neurodegenerative conditions, the link between the fragile X pre-mutation and parkinsonism, as expressed in the fragile X associated tremor and ataxia syndrome (FXTAS), is now well established [25–27].

In the present report, we systematically examine the relationship between parkinsonian signs and ASD in middle-aged and older adults. In study I, we conduct an exploratory study in 19 adults with ASD, over 49 years of age. In study II, we follow up a hypothesis generated from study I, systematically assessing 37 adults with ASD, over 39 years of age, for evidence of parkinsonian signs.

## Methods

The initial, pilot study was conducted at the University of North Carolina (Chapel Hill) (NC). A replication study was conducted at the University of Western Australia (Fremantle) (WA).

### Study I (NC)

#### Sample and ascertainment

Inclusion criteria for the study were (1) ≥50 years of age, (2) a clinical diagnosis of ASD, and (3) an informant able to provide historical information about the participant. DSM-5 diagnosis of ASD was confirmed by clinical best estimate (JP) after review of the Autism Diagnostic Interview (ADI) [28], the Autism Diagnostic Observation Schedule (ADOS) [29], and all available information from medical records. The Institutional Review Board (IRB) at the University of North Carolina (Chapel Hill) approved this study. All subjects or their legal guardians provided informed, written consent.

Ascertainment efforts were conducted through multiple sources throughout North Carolina. Attempts were made to contact 14,132 individuals with ASD through mailings to the Autism Society of North Carolina (*N* = 14,034), record review of the Treatment Education of Adults and Children with Autism and other Communication Handicaps (TEACCH) program seen over the last 40 years (*N* = 24 contacted), NC state residential facilities for adults with developmental disabilities (*N* = 24 contacted), the UNC Autism Registry of the Carolina Institute for Developmental Disabilities (*N* = 17 contacted), outpatient attendance at the University of North Carolina Hospitals and Clinics (*N* = 14 contacted), and a large general medical practice that specifically cares for adults with developmental disabilities living in “intermediate care facilities for individuals with mental retardation (ICFMR)”, in North Carolina (*N* = 9 contacted). Overall, 30 individuals with ASD or their guardians consented to participate in the study. Three individuals declined participation after the screening process, due to time constraints, and eight were excluded after enrollment based on insufficient evidence supporting a DSM5 diagnosis of ASD. The final sample completing the study consisted of 19 individuals meeting ASD criteria.

### Study II (WA)

#### Sample and ascertainment

Inclusion criteria for the study were (1) ≥40 years of age, (2) a clinical diagnosis of ASD, and (3) an informant able to provide historical information about the participant. DSM-5 diagnosis of ASD was confirmed by clinical best estimate (SES) after review of the ADI [28], the ADOS [29], and all available information from medical records. The South Metropolitan Health Service Human Research Ethics Committee approved this study. All subjects or their legal guardians provided informed written consent.

Subjects were recruited from two sources: (1) a list provided by the Disabilities Services Commission of Western Australia (DSC) which included 46 individuals 40 years or older with a diagnosis of ASD and (2) a list of 24 individuals with a diagnosis of ASD, 40 years or older, who had attended the Psychiatry Clinic at the Autism Association (AA) of Western Australia during the first 10 months of 2014. The DSC provides services to all registered individuals diagnosed with ASD in WA. The Psychiatry Clinic at the AA is the only clinic in WA specialized in assessing and managing challenging behaviors in adults with ASD. Individuals with ASD and/or their respective caregivers were contacted for participation. Twenty-nine of the 46 individuals from the DSC list consented to participate. All 29 completed the assessment protocol. Of the 17 individuals who did not participate in the study, 13 refused to participate or did not return a signed consent form, and four could not be contacted. Eleven of the 24 individuals with ASD from the AA Psychiatry Clinic list consented to participate, and eight completed the assessment protocol (assessments were not finalized on three subjects due to loss of contact or job commitments). Of the 13 individuals who did not participate, five refused or did not return a signed consent form, and eight could not be contacted.

### Assessment

All subjects and/or their informants were interviewed with a structured assessment for demographic information and medical history. Cognitive level (IQ group) was derived from scores available through medical records. When valid IQ estimates were not available, estimation of IQ group was based on administration of one of three measures of cognitive level or adaptive functioning: the Wechsler Abbreviated Scale of Intelligence [30], the Shipley-2 Scale [31], or the Vineland Adaptive Behavior Scales [32], depending on level of subject cooperation/ability. For the analyses, we collapsed cognitive scores into two groups (IQ ≥50 and <50). For study I, motor signs were assessed with the Unified Parkinson’s disease Rating Scale (UPDRS) [33] by research assistants (LP, MP) trained at the UNC Neurology Clinic by a board certified neurologist and expert in degenerative neurological disorders (Daniel Kaufer, M.D.) and supervised by a psychiatrist (JP). The UPDRS is a reliable and valid instrument for assessing the severity of motor signs associated with Parkinson’s disease. A limitation of the UPDRS for use in this population is that individuals with moderate to severe comprehension deficits may not be able to perform some of the tasks assessing bradykinesia (e.g., items 23 to 26) or their limited comprehension may make them unsuitable for valid testing of postural stability. Therefore, a modification was used whereby final clinical judgment on the presence of bradykinesia was based on scores rating limb bradykinesia (items 23, 24, 25, and 26) and/or scores on body bradykinesia and hypokinesia (item 31) (i.e., total bradykinesia was rated based on the presence of body bradykinesia only whenever assessment of limb bradykinesia was not feasible). For study II, motor signs were assessed with the Movement Disorders Society-Unified Parkinson’s disease Rating Scale (MDS-UPDRS) (an updated and expanded version of the UPDRS) by a neuropsychiatrist with extensive experience in assessing and diagnosing individuals with Parkinson’s disease (SES, a neuropsychiatrist with extensive experience in rating parkinsonism in complex patients, such as older adults with psychosis, dementia, or severe depression). As with the UPDRS, a limitation of the MDS-UPDRS for use in this population is that individuals with moderate to severe comprehension deficits may not be able to perform some of the tasks assessing bradykinesia (e.g., items 3.4 to 3.8) or postural stability. Therefore, a modification was employed whereby final clinical judgment on the presence of bradykinesia was based on scores rating limb bradykinesia (items 3.4 to 3.8) and/or scores rating global spontaneity of movement (item 3.14) (i.e., total bradykinesia was rated based on the presence of body bradykinesia only whenever assessment of limb bradykinesia was not feasible).

For both studies, motor signs were considered to be present whenever the participant scored 2 or higher on the respective UPDRS or MDS-UPDRS item. Thus, if a score of 2 or more was given to one or more of the items rating tremor, rigidity, bradykinesia, or postural problems, the sign was rated as present. Scores of “1” (i.e., slight or mild) were considered absent (and collapsed with scores of “0”). Diagnosis of parkinsonism was made using the Queen Square Brain Bank Criteria for idiopathic Parkinson’s disease, based on UPDRS findings [34]. We followed step 1 of the diagnostic criteria for “Parkinsonian syndrome” which requires the presence of bradykinesia and at least one of the following: (1) rigidity, (2) 4–6-Hz resting tremor, or (3) postural instability.

## Statistical analysis

Statistical analysis was carried out using means and standard deviations and *t* tests for between-group comparisons. Associations appearing in frequency distributions were tested using a Chi-square test (with Yates’ correction for cell sizes <5). All *p* values are two-tailed, with the alpha value equal to 0.05. Chlorpromazine equivalent estimates were calculated based on the Woods algorithm [35].

## Results

### Study I

Study I included 19 males, with an average age of 57 years. Eleven (58 %) had IQ’s below 50; nine (47 %) lived in residential facilities for adults with developmental disabilities, four (19 %) lived in-group homes, four lived independently with support, and one (5 %) lived independently in the community (Table 1).

**Table 5:** To provide additional descriptive data on the frequency of combined parkinsonian signs within individuals, below, we list individuals by the various combinations of parkinsonian signs

	Number of subjects	
Signs present	Study 1	Study 2
Tremor + rigidity + bradykinesia + postural instability	0	1
Tremor + rigidity + bradykinesia	1	3
Postural instability + bradykinesia	1	5
Tremor + bradykinesia	0	2
Rigidity + bradykinesia	0	1
Postural instability + rigidity	1	0
Tremor + rigidity	1	1
Rigidity only	2	1
Bradykinesia only	1	5
Tremor only	1	1
Postural Instability only	1	0

The frequency of occurrence of the cardinal signs for parkinsonism were 22 % (*N* = 4) with bradykinesia, 16 % (*N* = 3) with resting tremor, 32 % (*N* = 6) with rigidity, and 15 % (*N* = 2) with postural instability (Table 2). Assessments of postural instability and bradykinesia were difficult to conduct in six subjects and one subject, respectively, and these ratings were not further considered. There were no significant group differences between subjects in the higher and lower IQ groups (i.e., less than or ≥50) on resting tremor (*χ*^2^ = 2.59, df = 1, *p* = 0.11), rigidity (*χ*^2^ = 2.17 = df = 1, *p* = 0.14), bradykinesia (*χ*^2^ = 0.06, df = 1, *p* = 0.80), or postural instability (*χ*^2^ = 0.13, df = 1, *p* = 0.72) (Table 3). No significant differences were noted between subjects currently on atypical neuroleptics (referred to as “neuroleptics”) (*N* = 7) compared to those not on neuroleptics (*N* = 12) on the presence of resting tremor (*χ*^2^ = 0.02, df = 1, *p* = 0.89), rigidity (*χ*^2^ = 0.65, df = 1, *p* = 0.42), bradykinesia (*χ*^2^ = 0.42, df = 1, *p* = 0.52), or postural instability (*χ*^2^ = 0.71, df = 1, *p* = 0.40) (Table 4; Table 5).

**Table 1:** Demographics

	Study I (NC)	Study II (WA)
Sample size/gender	19 males	32m/5f
Age: mean (SD) years	57 (6.7)	51.2 (8.5)
Range years	50–77	40–71
Education		
No school/grade school	47 %	92 %
High school or equivalent	47 %	8 %
Post high school	6 %	0 %
IQ group		
≥80	21 %	5 %
50–79	21 %	14 %
35–49	16 %	14 %
≤35	42 %	67 %
Living situation		
Independent	5 %	11 %
Independent with support	21 %	0 %
With family (parents)	5 %	8 %
Group home	21 %	81 %
Residential facility	47 %	0 %

**Table 2:** Frequency of Parkinsonian signs

Motor signs	Study I	Study II
	% (*N*)	% (*N*)
Resting tremor	16 (3)	19 (7)
Rigidity	32 (6)	19 (7)
Bradykinesia	22 (4)^a^	46 (17)
Postural instability	15 (2)^a^	19 (7)

^a^One subject in study I could not be assessed for bradykinesia (total available *N* = 18); six subjects in study I could not be assessed for postural instability (total available *N* = 13)

**Table 3:** Frequency of Parkinsonian signs by IQ group

Motor signs % (*N*)	Study I		Study II	
	IQ group		IQ group	
	≥50 (*N* = 8)	<50 (*N* = 11)	≥50 (*N* = 7)	<50 (*N* = 30)
Resting tremor	0 (0)	27 (3)	29 (2)	17 (5)
Rigidity	50 (4)	18 (2)	29 (2)	17 (5)
Bradykinesia	25 (2)	20 (2)^a^	43 (3)	57 (17)
Postural instability^a^	13 (1)	20 (1)^a^	14 (1)	20 (6)

^a^One subject, with an IQ score less than 50, in study I could not be assessed for bradykinesia (total available *N* = 10); six subjects with IQ scores less than 50 in study I could not be assessed for postural instability (total available *N* = 5)

**Table 4:** Frequency of Parkinsonian signs by current neuroleptic treatment

Motor signs % (*N*)	Current medication treatment			
	Study I		Study II	
	Yes (*N* = 7)	No (*N* = 12)	Yes (*N* = 29)	No (*N* = 8)
Resting tremor	14 (1)	17 (2)	24 (7)	0
Rigidity	43 (3)	25 (3)	24 (7)	0
Bradykinesia	14 (1)	27 (3)^a^	48 (14)	38 (3)
Postural instability	0^a^	20 (2)^a^	17 (5)	25 (2)

^a^One subject who does not currently take neuroleptic medication in study I could not be assessed for bradykinesia (total available *N* = 11), three subjects currently taking neuroleptic medication could not be assessed for postural instability (*N* = 4), and three subjects who are not currently taking medication in study I could not be assessed for postural instability (total available *N* = 9)

Three of the 19 subjects (16 %) met the criteria for parkinsonism. Twelve of the 19 subjects (63 %) were not on neuroleptic medication. Two subjects (17 %) also had an independent (no knowledge of aims or goals of this study) diagnosis of Parkinson’s disease made by a community-based neurologist. One was on levodopa treatment at the time of the assessment and the second was recommended to take levodopa (see description in Appendixes 1 and 2).

### Study II

Study II included 32 males and 5 females, with an average age of 51 years. Thirty subjects (81 %) had an IQ below 50. Thirty-one (84 %) lived in a group home, four (11 %) lived independently in the community, and three (8 %) lived with their families (Table 1).

The frequency of occurrence of the cardinal signs for parkinsonism were 46 % (*N* = 17) with bradykinesia, 19 % (*N* = 7) with resting tremor, 19 % (*N* = 7) with rigidity, and 19 % (*N* = 7) with postural instability (Table 2). There were no significant group differences between subjects in the higher and lower IQ groups (i.e., less than or ≥50) on resting tremor (*χ*^2^ = 0.52, df = 1, *p* = 0.46), rigidity (*χ*^2^ = 0.52, df = 1, p = 0.46), bradykinesia (*χ*^2^ = 0.01, df = 1, *p* = 0.98), or postural instability (*χ*^2^ = 0.12, df = 1, *p* = 0.72) (Table 3). There were no significant differences between subjects on neuroleptics (*N* = 29) as compared to those not on neuroleptics (*N* = 8) on presence of resting tremor (*χ*^2^ = 2.38, df = 1, *p* = 0.12), rigidity (*χ*^2^ = 2.38, df = 1, *p* = 0.12), bradykinesia (*χ*^2^ = 0.29, df = 1, p = 0.58), or postural instability (*χ*^2^ = 0.24, df = , *p* = 0.61) (Table 4).

Twelve of the 37 subjects (32 %) met the diagnostic criteria for parkinsonism. Twenty-nine of the 37 subjects (78 %) were currently on neuroleptic medication. Ten of the 29 subjects on neuroleptics (34 %) met the criteria for parkinsonism. Eight of the 37 subjects (22 %) were not taking neuroleptic medication. Two (25 %) of these eight subjects met the criteria for parkinsonism. During the course of the study, these two individuals were referred to the Movement Disorders Clinic at Fremantle Hospital due to motor problems impacting upon their ability to complete basic activities of daily living. An independent diagnosis of Parkinson’s disease was made in both subjects by a community neurologist (see description in the Appendixes 1 and 2). Given the substantial number of subjects receiving neuroleptic medications, we examined group differences in level of chlorpromazine equivalents (an algorithm based on the potency of a specific medication and dose) [35] in those currently taking and not taking these medications. There were no significant differences in mean level of chlorpromazine equivalents between subjects classified with and without Parkinsonism-1 (mean mg ± SD: no PS-1 = 474 ± 758; PS-1 = 246 ± 220; *t* = 1.01, df = 35, *p* = 0.31).

### Studies I and II

Given the small number of subjects not currently on neuroleptic medication in each study, samples were combined (*N* = 56) with the aim of providing the best estimate of the rate of parkinsonism in this adult ASD population. The mean age of the combined sample of the 20 subjects not on neuroleptics was 55.6 ± 8.6 (years ± SD), range 42–77 years. All subjects were male. Four subjects (20 %) received a diagnosis of Parkinson’s disease by independent neurologists (*N* = 3) or were started on levodopa (*N* = 1). Available information documented that none of these four subjects had been given a neuroleptic medication in the previous 5 years (and perhaps longer, but sufficient documentation was not available to make a statement about a longer period of time in all four subjects). There was no significant difference in rate of parkinsonism (4/20 subjects, or 20 %) in this group versus the 11/36 subjects (31 %) from the combined study group who were on neuroleptics (*χ*^2^ = 0.52, df = 1, *p* = 0.46). A significantly greater number of subjects were on neuroleptics in study II (78 %) than in study I (37 %) (*χ*^2^ = 7.71, df = 1, *p* = 0.006).

## Discussion

In this paper, high rates of parkinsonism are reported in a sample of 19 adults with ASD over 49 years of age, who were directly examined, characterized on a structured rating scale, and diagnosed according to the standardized diagnostic criteria. These findings were then replicated in an independent sample of 37 adults with ASD over age 39. After excluding subjects currently on neuroleptic medications, the frequency of occurrence of parkinsonism was 20 % in the combined sample and 17 and 25 %, in each sample, respectively. Four patients (7 % of the combined sample) were diagnosed with PD by community neurologists. In two patients, the diagnosis should be considered tentative—one was on levodopa and while they met the criteria for parkinsonism, they had no explicit diagnosis of PD in the medical record, whereas the second patient met the diagnostic criteria for parkinsonism and was diagnosed as having “lower limb parkinsonism.”

The findings from this study suggest high rates of parkinsonism among ASD individuals over 39 years of age. The rate of parkinsonism in the general population aged 65–70 has been estimated at 0.9 % [36], as compared to 27 % in our combined total sample and 20 % when subjects on atypical neuroleptics were excluded. It is important to note that the de Rijk et al. [36] study included individuals with drug-induced parkinsonism in their parkinsonism group, making the comparison even more conservative (as those subjects were excluded from the current study). Moreover, in the current study, parkinsonism was only diagnosed when it occurred together with bradykinesia (a required criterion), whereas in the de Rijk et al. [36] study, parkinsonism was diagnosed if any two of the four cardinal symptoms for PD were present. Furthermore, the rates observed in the present study were considerably higher than the 0.1 % published rate for Parkinson’s disease in the general population 40 to 60 years of age [37].

The estimates in the current study are conservative in that, in order to maximize accuracy in classifying affected individuals, we used a cutoff of ≥2 when rating the UPDRS and MDS-UPDRS and ratings of 1 were collapsed with ratings of “0” (i.e., rated as “absent”). We view this as a conservative approach that may have led to several false negative classifications (see Appendixes 1 and 2 for clinical examples and further clarification). In addition, rates of parkinsonism were based on the entire sample of subjects in each study group, although a substantial portion of subjects in both groups were unable to be rated on signs that required active participation (e.g., finger tapping to rate bradykinesia and postural instability among non-verbal subjects).

The observation that the frequency of motor signs did not differ between those subjects currently taking neuroleptic medications versus those not on such medications, as well as the observation that parkinsonism was undiminished in several subjects on successively lowered doses of atypical neuroleptics, suggests the possibility that some cases of parkinsonism, observed in individuals on neuroleptics, may have been misclassified as being secondary to medications, when in fact they may have had a primary parkinsonism. In the Appendixes 1 and 2, we describe motor signs in a subject whose motor abnormalities were not improved on decreasing and ultimately discontinued (for 4 months as of the time of this submission) doses of olanzapine. In conclusion, it is highly likely that we may have under-estimated the true frequency of both PD and parkinsonism in ASD, which may be higher than herewith reported.

The process of diagnosing Parkinson’s disease in ASD is complex for several reasons: (1) As we found in this study, many adults with ASD are or have been on neuroleptic medication. (2) ASD has its own motor idiosyncrasies, such as motor stereotypies, generic “clumsiness,” and dyspraxia, which complicate the neurological exam. (3) Non-verbal ASD subjects are difficult to assess with the UPDRS/MDS-UPDRS, given that some of them are unable to follow commands. (4) Given that many adults with ASD live in-group homes or other supported accommodations, with a high turnover of caregivers, information about whether parkinsonian signs are progressive, with persistent asymmetry or had a unilateral onset, cannot always be ascertained. This limitation makes it difficult to use step 3 criteria of the UK Parkinson’s disease Society Brain Bank Clinical Diagnostic Criteria for a definitive diagnosis of Parkinson’s disease. We provide clinical vignettes in the Appendixes 1 and 2 that illustrate several of the complexities we encountered in our efforts to classify these individuals relative to the presence or absence of Parkinson’s disease.

An important limitation of this set of studies was the absence of local control groups for direct comparison of rates of parkinsonism and Parkinson’s disease. The lack of a systematic ascertainment scheme in study I precluded the identification of a suitable comparison group. While ascertainment in study II was done more systematically, lack of sufficient funding for that study precluded the addition of a comparably ascertained and assessed contrast group. Nevertheless, we believe that there is no evidence that either study was biased towards recruiting subjects with movement disorders, and the comparable rates observed in the two independent samples examined raises confidence in the validity of the findings. In addition, there was no evidence that potential confounding factors (e.g., low IQ or living in a long-term residential care facility) explained the rates of parkinsonism observed, as rates in the high and low IQ groups and between those living in residential care and others did not differ significantly.

The proportion of subjects with low IQ in both studies was considerably higher than current population estimates based on epidemiologically ascertained samples of school-aged children with autism [5]. A likely reason for this is that the rates of low IQ in the current sample are a reflection of ascertainment characteristics in childhood autism samples of the 1960s and 1970s, when many of the subjects in this study were diagnosed. It is possible that many higher functioning children with autism in the 1960s and 1970s were either undetected or misdiagnosed and continue to be under-recognized as adults, resulting in the difficulties we encountered in finding adults with ASD over 50 in the North Carolina sample and the higher proportion of subjects with low IQ ascertained in both study groups. This possibility is supported by findings from Mandell et al. [38], where 10 % of adults with autism in a state psychiatric hospital were found to be misdiagnosed with schizophrenia and other psychiatric conditions. Finally, accurate calculation of IQ scores in adults with ASD was an additional problem encountered in this study. IQ was estimated in some cases, from the Vineland adaptive behavior composite. Given that most of these participants live in-group homes with restrictive policies regarding activities (e.g. cooking, traveling independently), individuals with higher capacity and skills may have been inadvertently penalized with relatively lower scores.

While the findings from these two studies suggest a relationship between ASD and Parkinson’s disease, we can only speculate on the underlying explanation for this observed relationship. Several possibilities exist. First, given the relatively small size and the lack of a population-based ascertainment of both study groups, an unknown ascertainment bias must be considered as a possible explanation. Second, while current medication use did not appear to explain the rates of parkinsonism in either study, it is possible that long-term neuroleptic use and an underlying increased sensitivity to those medications may have had the effect of increasing the risk for parkinsonism later in life (even in those now off such medications). Thus, future studies should assess lifetime exposure to neuroleptic medication and examine the possibility that individuals with ASD, exposed to neuroleptics during their lifetime, may be at increased risk of developing Parkinson’s disease in mid- and later-life. A third possibility is that neuroleptic use may have “unmasked” a pre-clinical stage of Parkinson’s disease, perhaps lowering the threshold for dopamine loss in the development of Parkinson’s disease [39]. This hypothesis warrants examination in longitudinal studies of younger populations, as well as in older populations where better historical information is available.

Of greatest interest is a fourth possibility, that ASD and Parkinson’s disease share a common underlying pathogenetic mechanism or mechanisms that have both early and later manifestations. This idea is reminiscent of the early effects of the expanded triplet repeat in the FMR1 gene in causing both fragile X syndrome in children and the later effects of the FMR1 pre-mutation in causing fragile X-associated tremor and ataxia in carrier grandfathers in their fifth and sixth decades of life [25]. Parkinson’s disease may itself be a syndrome with multiple etiologies, some of which may be shared with ASD.

The findings from these studies, linking ASD and PD, have potential implications for understanding the neurobiology of both conditions. Links between the two have been made previously based on overlapping phenomenology and neurobiology [8]. Motor stereotypies are a well-known feature of ASD in a subset of individuals with autism [40], and an emerging literature suggests that a variety of other types of motor deficits (e.g., postural control, precision grip, and motor learning) are present at high rates in individuals with autism [12–17, 41–43]). Making observations of motor impairment in siblings and twins concordant and discordant for autism, Hilton et al. [44] have suggested that motor impairment constitutes a core characteristic of autism and that it is influenced by genetic factors. Also of interest is the relatively recent observation in high risk infant studies that gross motor deficits in the first year of life appear to precede the appearance of social deficits in the latter part of the first and second years, in high risk infants who go on to develop ASD [19–21]. Motor deficits in infancy have also been observed in retrospective, videotape analyses of individuals with autism [18]. Together these findings suggest a more primary and fundamental role of motor dysfunction in the pathogenesis of autism.

Common biological underpinnings between autism and Parkinson’s disease have also been suggested. Consistent with the behavioral observations of deficits in motor function in autism, brain abnormalities have been reported in individuals with autism and mouse models of autism, in regions and structures linked to the motor system. Structural imaging studies have noted abnormalities in the striatum [9–11, 45] and resting state functional abnormalities in primary motor cortex [46]. The cerebellum has also been implicated as playing a role in Parkinson’s disease pathophysiology [47], is known to be interconnected to the basal ganglia [48], and has been noted to be abnormal in individuals with autism on postmortem examination [49, 50]; brain imaging [51, 52], rapid precision grip [53], and in mouse models of the disorder [54, 55]. Genetic links between PD and autism have also been suggested by molecular studies identifying a mutation in Parkinson disease-associated, G-protein-coupled receptor in a number of unrelated autistic individuals [24]. Finally, the finding of high rates of parkinsonism in pre-mutation carriers of the fragile X mutation who manifest signs of FXTAS [25–27] suggests a possible common underlying biology in the subset of individuals with autism who go on to have Parkinson’s disease in later life. The molecular signaling pathways involved in fragile X (e.g., phosphoinositide 3-kinase or PI3-kinase and mammalian target of rapamycin or mTOR) lay at the confluence of molecular signaling pathways underlying several other genetically defined autistic syndromes (e.g., Rett Syndrome, PTEN, and tuberous sclerosis) and have been implicated in Parkinson’s disease [56–58]. While specific biological mechanisms underlying the possible relationship between Parkinson’s disease and autism remain to be explored, identifying those individuals with parkinsonism in adulthood is a reasonable a strategy to explore in identifying a more etiologically homogenous subgroup with autism than those with idiopathic autism.

The possibility that individuals with autism are at increased risk for Parkinson’s disease as adults has important implications for detection and assessment, clinical practice, systems of care, training, and public policy. While research on ASD is now underway in young adult populations, the striking absence of research on autism in “older” adults has been noted in the literature [7]. This dearth of knowledge about the problems faced by this population is likely to have a serious impact on future quality of care, with the increasing demands for care associated with the dramatic aging of western societies. The finding of high rates of Parkinson’s disease in older adults with autism is likely to be only one of many potential new findings to be discovered, as the research community turns its attention to this relatively neglected population. Such findings will have important implications for building capacity to care for such individuals, as it is likely that there will be unique aspects of their care that will need to be appreciated. If the findings of the present study are confirmed, there will be a need to understand the underlying pathogenesis of parkinsonism and Parkinson’s disease in ASD, to examine co-occurring behavioral and other features of the condition and to explore potential differences in treatment from the traditional approaches to Parkinson’s disease in individuals without ASD. Our finding of a high frequency of parkinsonism among middle-age and older autistic individuals on atypical neuroleptics emphasizes the urgent need to clarify whether there is a subset of individuals with ASD at a high risk for developing movement disorders, as well as exploring the possibility that neuroleptic medications may unmask or precipitate true Parkinson’s disease in older individuals with ASD. As a more general observation, the vast majority of the subjects we detected who had parkinsonism and were on neuroleptic medications, had motor deficits not appreciated by their care providers or noted in the medical records. Failure to recognize drug-induced parkinsonism in the elderly is well described [59], and it is possible that recognition may be even lower in elderly with ASD. Co-morbid dementia may complicate a diagnosis of Parkinson’s disease, but none of our subjects had a history of functional decline or stepwise deterioration.

The lack of controls in both studies and ascertainment scheme in study I have been noted above as limitations of this work. There were also differences between studies in level of education, IQ, and living arrangements that may have had an unknown impact on the findings. Another limitation was that only 22 % of patients in study 2 were neuroleptic free, as compared to 63 % in study 1. These findings suggest that a relatively high percentage of adults with ASD will be on neuroleptic medication and/or will have a history of neuroleptic intake. This will be an important confounder for future studies aiming at ascertaining representative samples of adults with ASD off neuroleptics. The safety of withdrawal of neuroleptics among ASD individuals with well-controlled challenging behaviors remains unknown. A better option could be using DaTscan studies, a functional technique of imaging the dopamine transporter that helps to determine presynaptic dopamine neuronal degeneration [60]. DaTscan studies may help distinguishing neurodegenerative from drug-induced parkinsonism, although false-positive results in drug-induced parkinsonism has been reported [61]. Other limitations included the lack of establishment of inter-rater reliability within and across studies, although good reliability of the UPDRS and MDS-UPDRS has been demonstrated [33, 62], limited information on life-long medication history and, for a number of subjects, limited information about early childhood behavior (to provide more certainty of diagnosis). Our relatively small sample size may account for the lack of significant differences in frequency of parkinsonism between ASD individuals on vs. off neuroleptics, as well as the lack of significant association between parkinsonism and clinical variables such as IQ. In addition, while it seems unlikely to be an explanation of the findings from the present studies, there is a possibility that some affected subjects had fragile X syndrome co-occurring with their ASD, as the presence of the fragile X mutation is known to account for a small portion of cases of ASD, and the FMR1 pre-mutation has been linked to risk for Parkinsonian motor deficits [27, 63]. Future studies of Parkinson’s disease and parkinsonism in older adults with ASD should rule out the presence of the fragile X mutation in their study subjects.

## Conclusions

We find a high frequency of parkinsonism among ASD individuals older than 39 years. If high rates of parkinsonism and potentially Parkinson’s disease are confirmed in subsequent studies of ASD, this observation has important implications for understanding the neurobiology of autism and treatment of manifestations in older adults. Given the prevalence of autism in school-age children, the recognition of its life-long natural history, and the recognition of the aging of western societies, these findings also support the importance of further systematic study of other aspects of older adults with autism.

## Appendix

To maximize confidentiality, demographic information was not included in the vignettes below. For the following vignettes, we employ the following scoring convention: (1) UPDRS: 0 = absent; 1 = mild; 2 = moderate; 3 = severe, 4 = unable to perform task; (2) MDS-UPDRS: 0 = absent, 1 = slight, 2 = mild, 3 = moderate, 4 = severe.

### Description of ASD individuals meeting diagnostic criteria for parkinsonian syndrome

#### Study I

##### Subject 1

On the UPDRS, this subject had moderate resting and action tremor in both upper limbs; moderate rigidity in the right upper limb, marked rigidity in the left upper limb, and severe rigidity in both lower limbs; marked bilateral bradykinesia on hand movements; moderate deficit on arising from chair; moderately stooped posture; shuffling gait; mild postural instability; and moderate body bradykinesia (note: finger tapping, rapid alternating movements, and leg agility could not be assessed due to poor comprehension/cooperation). Total UPDRS motor score was 38 (note: total score refers to the cumulative score across individual items appearing in section 3 of the UPDRS/MDS-UPDRS). Subject 1 was on carbidopa/levodopa 250 mg t.i.d started by a neurologist independent of the study.

##### Subject 2

On the UPDRS, this subject showed mild resting tremor in the right upper limb; mild rigidity of the neck and the right lower limb; mild stooped posture; marked shuffling gait; marked absence of postural reflexes; and moderate body bradykinesia (note: hand movements and rapid alternating movements could not be tested due to poor comprehension/cooperation). Total UPDRS motor score was 12. An independent neurology report indicated that symptoms were consistent with “gait predominant Parkinsonism” and that a trial treatment with Sinemet “might be beneficial.” The subject was not receiving this treatment at the time of the study.

#### Study II

##### Subject 3

On the MDS-UPDRS, this subject showed slight bilateral kinetic tremor, slight bilateral bradykinesia on finger tapping, mild bradykinesia on hand movements in the right upper limb, and moderate bradykinesia of the left upper limb. He had slight bradykinesia on pronation-supination of the right hand and moderate bradykinesia of the left hand, slight bilateral bradykinesia on both toe tapping and leg agility; mild shuffling gait; absence of postural response; moderate stooped posture; and mild body bradykinesia. Total MDS-UPDRS motor score was 28. This individual was diagnosed with Parkinson’s disease by a neurologist independent of the study. He was recently started on levodopa (100 mg tds) with marked improvement on upper limb tremor.

##### Subject 4

On the MDS-UPDRS, this subject showed mild bilateral bradykinesia on finger tapping, hand movements, pronation-supination movements of hands, toe tapping, and leg agility; slight slowness arising from a chair; slight shuffling gait; absence of postural response; and mild body bradykinesia. Total MDS-UPDRS motor score was 28. This individual was diagnosed with Parkinson’s disease by a neurologist independent of the study but was not on levodopa at the time of the assessment.

### Possible false negative classification

We conservatively considered scores of 1 on the UPDRS/MDS-UPDRS (i.e., slight or mild) as 0 (i.e., “absent”). The rationale for this was to increase certainty in ratings of motor signs deemed to be “present” and concerns about reliability of ratings of “1.” However, we believe in some cases this may have led to false negative classification. Specifically, several subjects not on neuroleptics were rated a 1 on items involving multiple measurements (such as “rigidity” involving multiple limbs) or had evidence of cardinal signs that were measured in multiple ways (e.g., multiple items for bradykinesia), but all ratings were 1. Regardless of the consistency across items or measurements within a single domain, a rating of 1 was always rated as equivalent to “absent”. An example is provided below:

#### Subject A

On the UPDRS, this subject showed severe neck rigidity, marked rigidity in the right upper and lower limbs, and slight rigidity on the left lower limb. He was also rated as having mild bilateral bradykinesia in the upper limbs on rapid alternating movements, mild slowness in arising from a chair, slightly stooped posture, moderate difficulty in walking due to a shuffling gait, and mild postural instability. Total UPDRS motor score was 21.

### ASD subject with parkinsonian syndrome, on neuroleptics at the time of assessment

The subject below met criteria for Parkinsonian syndrome; but was on neuroleptic medication (olanzapine 7.5 mg/day).

#### Subject B

On the MDS-UPDRS, this subject showed mild postural, kinetic, and resting tremor of the right upper limb and moderate postural, kinetic, and resting tremor of the left upper limb; mild rigidity in the neck, both upper limbs, and the right lower limb; mild bilateral bradykinesia on finger tapping, hand movements, pronation-supination of hands, toe tapping, and leg agility; slight slowness on arising from a chair; slight shuffling gait; absence of postural response; moderate stooped posture; and moderate body bradykinesia. Total UPDRS motor score was 56. Olanzapine was slowly reduced and ceased, but at 4-month follow-up, parkinsonian signs remained unchanged.

### Scoring system

*Tremor* assessment is divided into (1) postural tremor of the hands and (2) kinetic tremor of the hands (which is tested by the finger-to-nose maneuver) and rest tremor amplitude (this includes tremor that may appear at any time during the exam, including when quietly sitting, during walking, and during other bodily movements). Score range from normal (score = 0) to severe (score = 4).

*Rigidity* is judged on slow passive movement of major joints with the individual in a relaxed position and the examiner manipulating the limbs and neck. Neck and limbs are tested and rated separately, and scores range from normal (score = 0) to severe (score = 4).

*Bradykinesia* is tested on finger tapping, hand movements, pronation-supination movements of hands, toe tapping, leg agility, and global spontaneity of movement (body bradykinesia). Each limb is tested and rated separately evaluating speed, amplitude, hesitations, halts, and decrementing amplitude. Body bradykinesia provides a score combining all observations on slowness, hesitancy, and small amplitude and poverty of movement in general. Scores range from normal (score = 0) to severe (score = 4).

*Postural stability* is the examination of the response to sudden body displacement produced by a quick, forceful pull on the shoulders while the individual is standing erect with eyes open and feet comfortably apart and parallel to each other. Scores range from normal (score = 0) to severe (score = 4).

## References

[1] American Psychiatric Association (2013). Diagnostic and statistical manual of mental disorders.

[2] Kats D, Payne L, Parlier M, Piven J (2013). Prevalence of selected clinical problems in older adults with autism and intellectual disability. J Neurodev Disord.

[3] Totsika V, Felce D, Kerr M, Hastings RP (2010). Behavior problems, psychiatric symptoms, and quality of life for older adults with intellectual disability with and without autism. J Autism Dev Disord.

[4] Geurts HM, Vissers ME (2012). Elderly with autism: executive functions and memory. J Autism Dev Disord.

[5] Developmental Disabilities Monitoring Network Surveillance Year 2010 Principal Investigators, Centers for Disease Control and Prevention (CDC) (2014). Prevalence of autism spectrum disorder among children aged 8 years—autism and developmental disabilities monitoring network, 11 sites, United States, 2010. MMWR Surveill Summ.

[6] United Nations (UN) (2002). World population ageing 1950–2050.

[7] Piven J, Rabins P (2011). Autism spectrum disorders in older adults: toward defining a research agenda. J Am Geriatr Soc.

[8] Hollander E1, Wang AT, Braun A, Marsh L (2009). Neurological considerations: autism and Parkinson’s disease. Psychiatry Res.

[9] Sears LL, Vest C, Mohamed S, Bailey J, Ranson BJ, Piven J (1999). An MRI study of the basal ganglia in autism. Prog Neuropsychopharmacol Biol Psychiatry.

[10] Estes A, Shaw DW, Sparks BF, Friedman S, Giedd JN, Dawson G, Bryan M, Dager SR (2011). Basal ganglia morphometry and repetitive behavior in young children with autism spectrum disorder. Autism Res.

[11] Langen M, Durston S, Staal WG, Palmen SJ, van Engeland H (2007). Caudate nucleus is enlarged in high-functioning medication-naive subjects with autism. Biol Psychiatry.

[12] Whyatt C, Craig C (2013). Sensory-motor problem in autism. Front Integr Neurosci.

[13] Ravizza SM, Solomon M, Ivry RB, Carter CS (2013). Restricted and repetitive behaviors in autism spectrum disorders: the relationship of attention and motor deficits. Dev Psychopathol.

[14] Liu T (2013). Sensory processing and motor skill performance in elementary school children with autism spectrum disorder. Percept Mot Skills.

[15] Nayate A, Tonge BJ, Bradshaw JL, McGinley JL, Iansek R, Rinehart NJ (2012). Differentiation of high-functioning autism and Asperger’s disorder based on neuromotor behavior. J Autism Dev Disord.

[16] Minshew NJ, Sung K, Jones BL, Furman JM (2004). Underdevelopment of the postural control system in autism. Neurology.

[17] Marko MK, Crocetti D, Hulst T, Donchin O, Shadmehr R, Mostofsky SH. Behavioural and neural basis of anomalous motor learning in children with autism. Brain 2015. http://dx.doi.org/10.1093/brain/awu394. First published online: 21 January 2015. 10.1093/brain/awu394 PMC4339776 25609685

[18] Teitelbaum P, Teitelbaum O, Nye J, Fryman J, Maurer RG (1998). Movement analysis in infancy may be useful for early diagnosis of autism. Proc Natl Acad Sci U S A.

[19] Bhat AN, Galloway JC, Landa RJ (2012). Relation between early motor delay and later communication delay in infants at risk for autism. Infant Behav Dev.

[20] Libertus K, Sheperd KA, Ross SW, Landa RJ (2014). Limited fine motor and grasping skills in 6-month-old infants at high risk for autism. Child Dev.

[21] Estes A, Zwaigenbaum L, Gu H, St. John T, Paterson S, Elison J, et al. Behavioral, cognitive, and adaptive development in infants with autism spectrum disorder in the first two years of life. J Neurodev Disord. 2015;7(1):24. Epub 2015 Jul 16. doi:10.1186/s11689-015-9117-6. 10.1186/s11689-015-9117-6 PMC4511527 26203305

[22] Glessner JT, Wang K, Cai G, Korvatska O, Kim CE, Wood S, Zhang H, Estes A (2009). Autism genome-wide copy number variation reveals ubiquitin and neuronal genes. Nature.

[23] Scheuerle A, Wilson K (2011). PARK2 copy number aberrations in two children presenting with autism spectrum disorder: further support of an association and possible evidence for a new microdeletion/microduplication syndrome. Am J Med Genet B Neuropsychiatr Genet.

[24] Fujita-Jimbo E, Yu ZL, Li H, Yamagata T, Mori M, Momoi T, Momoi MY (2012). Mutation in Parkinson disease-associated, G-protein-coupled receptor 37 (GPR37/PaelR) is related to autism spectrum disorder. PLoS ONE.

[25] Jacquemont S, Hagerman RJ, Leehey M, Grigsby J, Shang L, Brunberg JA, Greco C, Des PV, Jardini T, Levine R, Berry-Kravis E, Brown WT, Schaeffer S, Kissel J, Tassone F, Hagerman PJ (2003). Fragile X Premutation tremor/ataxia syndrome: molecular, clinical and neuroimaging correlates. Am J Hum Genet.

[26] Leehey MA, Berry-Kravis E, Goetz CG, Zhang L, Hall DA, Li L, Rice CD, Lara R, Cogswell J, Reynolds A, Gane L, Jacquemont S, Tassone F, Grigsby J, Hagerman RJ, Hagerman PJ (2008). FMR1 CGG repeat length predicts motor dysfunction in premutation carriers. Neurology.

[27] Hall DA, Howard K, Hagerman R, Leehey MA (2009). Parkinsonism in FMR1 premutation carriers may be indistinguishable from Parkinson disease. Parkinsonism Relat Disord.

[28] Lord C, Rutter M, Couteur A (1994). Autism diagnostic interview—revised: a revised version of a diagnostic interview for caregivers of individuals with possible pervasive developmental disorders. J Autism Dev Disord.

[29] Lord C, Rutter M, DiLavore PC, Risi S (2000). Autism diagnostic observation schedule.

[30] Wechsler D (1999). Wechsler abbreviated scale of intelligence.

[31] Zachary RA (1986). Shipley institute of living scale: revised manual.

[32] Sparrow S, Balla D, Cicchetti D (2005). Vineland adaptive behavior scales: second edition.

[33] Movement Disorder Society Task Force on Rating Scales for Parkinson’s disease (2003). The unified Parkinson’s disease rating scale (UPDRS): status and recommendations. Mov Disord.

[34] Hughes AJ, Daniel SE, Kilford L, Lees AJ (1992). Accuracy of clinical diagnosis of idiopathic Parkinson’s disease. A clinico-pathological study of 100 cases. JNNP.

[35] Woods SW (2003). Chlorpromazine equivalent doses for the newer atypical antipsychotics. J Clin Psychiatry.

[36] De Rijk MC, Tzourio C, Breteler MMB, Dartigues JF, Amaducci L, Lopex-Pousa S, Manubens-Bertran JM, Alperovitch A, Rocca WA, for the EUROPARKINSON Study Group (1997). Prevalence of parkinsonism and Parkinson’s disease in Europe: the EUROPARKINSON collaborative study. J Neurol Neurosurg Psychiatry.

[37] Wirdefeldt K, Adami HO, Cole P, Trichopoulos D, Mandel J (2014). Epidemiology and etiology of Parkinson’s disease: a review of the evidence. Eur J Epidemiol.

[38] Mandell DS, Lawer LJ, Branch K, Brodkin ES, Healey K, Witalec R, Johnson DN, Gur RE (2012). Prevalence and correlates of autism in a state psychiatric hospital. Autism.

[39] Thanvi B, Treadwell S (2009). Drug-induced parkinsonism: a common cause of parkinsonism in older people. Postgrad Med J.

[40] Langen M, Surston S, Kas MJH, van England H, Staal WG (2011). The neurobiology of repetitive behavior: and men. Neurosci Biobehav Rev.

[41] Wang Z, Magnon G, White SP, Greene R, Vaillancourt DE, Mosconi M (2014). Individuals with autism spectrum disorder (ASD) show abnormalities during initial and subsequent phases of precision gripping. J Neurophysiol.

[42] Hallett M, Lebiedowska MK, Thomas SL, Stanhope SJ, Dencla MB, Rumsey J (1993). Locomotion of autistic adults. Arch Neurol.

[43] Ament K, Mejia A, Buhlman R, Erklin S, Caffo B, Mostofsky S, Wodka E (2015). Evidence for specificity of motor impairments in catching and balance in Children with Autism. J Autism Dev Disord.

[44] Hilton CL, Zhang Y, Whilte MR, Klohr CL, Constantino J (2012). Motor impairment in sibling pairs concordant and discordant for autism spectrum disorders. Autism.

[45] Hollander E, Anagnostou E, Chaplin W, Esposito K, Haznedar MM, Licalzi E, Wasserman S, Soorya L, Buschbaum M (2005). Striatal volume on magnetic resonance imaging and repetitive behaviors in autism. Biol Psychiatry.

[46] Nebel MB, Joel SE, Muschelli J, Barber AD, Caffo BS, Pekar JJ, Mostofsky SH (2012). Disruption of functional organization within the primary motor cortex in children with autism. Hum Brain Mapp.

[47] Wu T, Hallett M (2013). The cerebellum in Parkinson’s disease. Brain.

[48] Bostan AC, Strick PL (2010). The cerebellum and basal ganglia are interconnected. Neuropsychol Rev.

[49] Skefos J, Cummings C, Enzer K, Holiday J, Weed K, Levy E, Yuce T, Kemper T, Bauman M (2014). Regional alterations in purkinje cell density in subjects with autism. PLoS ONE.

[50] Wegiel J, Flory M, Kuchna I, Nowicki K, Ma S, Imaki H, Wegiel J, Cohen IL, London E, Wisniewski T, Brown W (2014). Stereological study of the neuronal number and volume of 38 brain subdivisions of subjects diagnosed with autism reveals significant alterations restricted to the striatum, amygdala and cerebellum. Acta Neuropathol Commun.

[51] Jeong JW, Tiwari VN, Behen ME, Chugani HT, Chugani DC (2014). In vivo detection of reduced Purkinje cell fibers with diffusion MRI tractography in children with autistic spectrum disorders. Front Hum Neurosci.

[52] Hanaie R, Mohri I, Kagitani-Shimono K, Tachibana M, Azuma J, Matsuzaki J, Watanabe Y, Fujita N, Taniike M (2013). Altered microstructural connectivity of the superior cerebellar peduncle is related to motor dysfunction in children with autistic spectrum disorders. Cerebellum.

[53] Mosconi MW, Mohanty S, Greene RK2, Cook EH, Vaillancourt DE, Sweeney JA (2015). Feedforward and feedback motor control abnormalities implicate cerebellar dysfunctions in autism spectrum disorder. J Neurosci.

[54] Shpyleva S, Ivanovsky S, de Conti A, Melnyk S, Tryndyak V, Beland FA, James SJ, Pogribny IP (2014). Cerebellar oxidative DNA damage and altered DNA methylation in the BTBR T+tf/J Mouse model of autism and similarities with human post mortem cerebellum. PLoS ONE.

[55] Piochon C, Kloth AD, Grasselli G, Titley HK, Nakayama H, Hashimoto K, Wan V, Simmons DH, Eissa T, Nakatani J, Cherskov A (2014). Cerebellar plasticity and motor learning deficits in a copy-number variation mouse model of autism. Nat Commun.

[56] Timmons S, Coakley MF, Moloney AM, O’ Neill C (2009). Akt signal transduction dysfunction in Parkinson’s disease. J Neurosci Lett.

[57] Quesada A, Lee BY, Micevych PE (2008). PI3 kinase/Akt activation mediates estrogen and IGF-1 nigral DA neuronal neuroprotection against a unilateral rat model of Parkinson’s disease. Dev Neurobiol.

[58] Heras-Sandoval D1, Pérez-Rojas JM2, Hernández-Damián J1, Pedraza-Chaverri J (2014). The role of PI3K/AKT/mTOR pathway in the modulation of autophagy and the clearance of protein aggregates in neurodegeneration. Cell Signal.

[59] Esper CD, Factor SA (2008). Failure of recognition of drug-induced parkinsonism in the elderly. Mov Disord.

[60] Fang B, Martin W (2015). Dopamine transporter imaging as a diagnostic tool for parkinsonism and related disorders in clinical practice. Parkinsonism Relat Disord.

[61] Cilia R, Marotta G, Beletti A, Siri C, Pezzoli G (2014). Reversible dopamine transporter reduction in drug-induced parkinsonism. Mov Disord.

[62] Goetz CG, Tilley BC, Shaftman SR, Stebbins GT, Fahn S, Martinez-Martin P, Poewe W, Sampaio C, Stern MD, Dodel R, Dubois B, Hollaway R, Jankovic J, Kulisevsky J, Lang AE, Lees A, Leurgans S, LeWitt PA, Nyenhuis D, Olanow CW, Rascol O, Schrag A, Teresi JA, van Hilten JJ, LaPelle N, for the Movement Disorder Society UPDRS Revision Task Force (2008). Movement disorder society-sponsored revision of the unified Parkinson’s disease rating scale (MDS-UPDRS): scale presentation and clinimetric testing. Mov Disord.

[63] Niu Y-Q, Yang J-C, Hall DA, Leehey MA, Tassone F, Olichney JM, Hagerman RJ, Zhang L (2014). Parkinsonism in fragile X-associated tremor/ataxia syndrome (FXTAS): revisited. Parkinsonism Relat Disord.

